# Tubulointerstitial inflammation in glomerular diseases: mechanistic pathways, prognostic value, and translational therapeutic targets

**DOI:** 10.1080/0886022X.2026.2646426

**Published:** 2026-03-24

**Authors:** Zhi-liang Jiang, Yi-dong Zhou, Ru-jie Lin, Yue-ming Liu, Bin Zhu

**Affiliations:** ^a^ The Second School of Clinical Medicine, Hangzhou Normal University, Hangzhou, Zhejiang, China; ^b^ Urology & Nephrology Center, Department of Nephrology, Zhejiang Provincial People’s Hospital, Affiliated People’s Hospital, Hangzhou Medical College, Hangzhou, Zhejiang, China

**Keywords:** Tubulointerstitial inflammation, Glomeluar disease, Glomerular tubulointerstitial crosstalk, Chronic kidney disease

## Abstract

Tubulointerstitial inflammation (TII) serves as a critical nexus connecting glomerular injury, interstitial fibrosis, and kidney function decline, and is commonly observed across various glomerular diseases. Pathogenic drivers and crosstalk along the glomerulus–tubule–interstitium axis promote a persistent interstitial inflammatory microenvironment, wherein chemokines, cytokines, and adhesion molecules activate specific pathways to recruit immune cells. Despite increasing attention, the independent prognostic significance of TII remains inconsistent across different diseases and populations. Moreover, glomerulus-centric therapies have failed to halt disease progression in a substantial subset of patients, highlighting the limitations of strategies focused solely on the glomerulus. Methodological variations – such as denominator definitions (total cortex vs. non-fibrotic cortex), scoring systems (percentage-based vs. count/semi-quantitative methods), and inconsistent covariate adjustment – largely account for these discrepancies. This review systematically synthesizes current evidence on the pathogenic mechanisms, prognostic relevance, and translational limitations of TII in glomerular diseases. We further highlight emerging advances in AI-augmented digital pathology, spatial transcriptomics, noninvasive biomarkers, and imaging modalities, which collectively enable more refined spatial and quantitative characterization of interstitial immune–fibrotic niches. Finally, we advocate a conceptual shift from a glomerulus-centric framework toward a glomerulo-interstitial systems perspective, emphasizing standardized quantification, integrative modeling, and external validation as prerequisites for precision medicine approaches. Understanding the glomerulo-interstitial inflammatory axis not only refines CKD prognostication but also delineates therapeutic targets for precision nephrology, bridging pathology with clinical intervention.

## 1. Introduction

Chronic kidney disease (CKD) is a growing global public health concern, with glomerular disease recognized as a major etiological factor contributing to CKD and end-stage renal disease (ESRD). Epidemiological surveys indicate that approximately 10% of adults worldwide are affected by CKD [1], with a reported prevalence of 8.2% in the Sixth Chinese Chronic Disease and Risk Factor Surveillance [2]. Primary glomerulonephritis is the most common cause of CKD across all regions of China, with IgA nephropathy and membranous nephropathy being the predominant subtypes. Lupus nephritis is the most common secondary glomerulonephritis [3]. Although treatments targeting glomerular injury have been effective in reducing proteinuria in some CKD patients, such as immunosuppressants, angiotensin-converting enzyme inhibitors (ACEIs), and angiotensin receptor blockers (ARBs), a substantial proportion still progress to ESRD. This highlights inherent limitations in traditional ‘glomerulocentric’ treatment approaches.

Recent studies have shown that the progression of glomerular disease depends not only on intrinsic glomerular cell injury but also on the activation of renal interstitial inflammation [4]. Renal interstitial inflammation is characterized by immune cell infiltration (e.g., macrophages, T lymphocytes), the release of pro-inflammatory factors (e.g., TNF-α, IL-6), and dysregulation of fibrotic signaling pathways (e.g., TGF-β/Smad), which has been identified as an independent risk factor for renal function decline. Previous studies have demonstrated that interstitial lesions, including fibrosis and inflammation, possess stronger prognostic value than glomerular lesions [5]. As interstitial inflammation generally precedes irreversible fibrosis, it represents a critical and potentially modifiable phase for therapeutic intervention.

A major therapeutic challenge is that interventions targeting the glomerulus (e.g., ACEIs, B-cell-targeting agents, and complement inhibitors) exert only limited effects on interstitial inflammation, despite reducing upstream causative factors [6–8]. In recent years, new technologies such as single-cell sequencing and spatial multi-omics have enabled analysis of the renal interstitium’s heterogeneity and pathological mechanisms. These technologies have also promoted targeted research on key signaling pathways, including Janus kinase/Signal transducer and activator of transcription (JAK/STAT), the NOD-like receptor family pyrin domain containing3(NLRP3) inflammasome, and C-C motif chemokine ligand/C-C motif chemokine receptor (CCL2/CCR2). In parallel, prognostic biomarkers of interstitial inflammation, such as urinary N-acetyl-β-D-glucosaminidase (NAGL) [9] and urinary CD163 [10,11] are under investigation. More recently, advances in digital pathology, artificial intelligence–augmented whole-slide image analysis, and quantitative imaging have created new opportunities to spatially resolve interstitial immune–fibrotic niches and to integrate molecular, morphologic, and clinical dimensions of disease [12,13].

In this review, we comprehensively summarize the pathogenic mechanisms, clinical implications, and translational therapeutic strategies related to tubulointerstitial inflammation in glomerular diseases. Beyond conventional pathway-based interpretations, we incorporate emerging evidence from AI-assisted pathology, imaging-derived phenotyping, and integrated multi-omics approaches, and outline future directions for risk stratification and therapeutic targeting along the glomerular–tubulointerstitial axis. By doing so, we aim to move beyond a solely glomerular-centered framework toward a more integrated glomerular–tubule-interstitium model that better reflects disease progression and informs clinical management in nephrology.

## 2. The significance of interstitial inflammation in glomerular diseases

### 2.1. IgA nephropathy

Tubulointerstitial inflammation, characterized by the infiltration of monocytes/macrophages and T and B lymphocytes into the interstitium, is a hallmark histopathologic feature of IgAN. Multiple studies demonstrate that higher densities of interstitial T cells, macrophages, DC-SIGN^+^ dendritic cells, and mast cells are closely correlated with more severe clinical and pathologic phenotypes, including heavier proteinuria, faster eGFR decline, greater interstitial fibrosis/tubular atrophy, and more pronounced glomerular lesions [14–18]. Mechanistically, mesangial deposition of IgA immune complexes activates the NLRP3 inflammasome and the complement cascade, leading to inflammatory injury in glomerular cells [19]. Downstream release of damage-associated mediators and filtered proteins is sensed by tubular epithelial cells, which upregulate chemokines and adhesion molecules, recruit leukocytes into the interstitium, and amplify local inflammation [20–22]. These processes may be driven directly by mesangial cytokine release and indirectly through podocyte injury, ultimately establishing a ‘glomerulo–tubular–interstitial’ amplification loop.

Although total cortical interstitial inflammation correlates strongly with interstitial fibrosis, early efforts to score inflammation separately in scarred versus non-scarred regions showed poor reproducibility (intraclass correlation coefficient [ICC] = 0.03) [23]. This is the reason that interstitial inflammation was not directly incorporated into the Oxford MEST-C classification. To reassess its prognostic value, Myllymäki et al. suggested that interstitial inflammation may predict IgAN progression after adjustment for covariates including fibrosis. Seeking a more robust metric, Alastair J. Rankin and colleagues estimated active tubulointerstitial inflammation in non-atrophic cortex by subtracting the percentage of atrophic cortex from the percentage of cortex with tubulointerstitial inflammation. This measure significantly correlated with renal outcomes and emerged as an important predictor of progression in IgAN [24]. Complementing this, Zhu et al. scored total cortical interstitial inflammation in a dual-center cohort, and confirmed its reproducibility as a pathologic parameter. A higher inflammatory burden was associated with faster eGFR loss and increased risk of kidney failure. Incorporating this measure into the international IgAN risk tool improved risk discrimination, although the increase in AUC was modest and requires external validation [25]. In that study, two pathologists independently reviewed all fields on full-section slides and graded separately from interstitial fibrosis, edema, and tubulitis. Disagreements were resolved through joint review to minimize bias from unevenly affected areas. Aiello et al. performed a study that stratified treatment at diagnosis based on histologic activity and clinical status found that among patients with proteinuria >1 g/day and/or hypertension plus active lesions or marked interstitial inflammation, adding glucocorticoids to RASi for 6 months significantly mitigated the adverse effect of increased interstitial macrophages on outcomes [18].

Several studies investigated the effects of subgroups of interstitial inflammatory cells in IgAN, Greater interstitial infiltration of CD68+ macrophages is associated with worse renal outcomes and correlates with Oxford MEST-C parameters (particularly the T) [26]. Ever in C4d-negative biopsies, increased macrophage infiltration signals a higher risk of kidney failure [27]. Subtype analyses reveal phenotype divergence: M2a macrophages are linked to more severe interstitial pathology and functional impairment, whereas abundance of M2c macrophages inversely correlates with glomerulosclerosis and interstitial fibrosis [28]. Among T cells, higher densities of GMP17T + cells, FOXP3+ Tregs, and CD3+ T cells in the tubulointerstitium predict renal function decline [29–31]. In pediatric IgAN, increased interstitial CD8+ T cells indicate a higher risk of progression [32]. Although B cells are generally less abundant, their increase is still significantly associated with renal functional deterioration [33]. DC-SIGN+ dendritic cells correlate positively with infiltrates of CD68+, CD4+/CD8+, and CD20+ cells and, collectively track with disease progression – supporting a coordinated local immune network. Mast cells contribute to fibrogenesis [34] and may predict poor outcomes [35]. In a trial involving 736 high-risk IgAN patients (with persistent proteinuria ≥1 g/day despite at least 3 months of optimized RASi), tubulointerstitial CD68+ infiltration showed a statistically significant – through limited – association with response to immunosuppression [36]. Collectively, these findings delineate a multicellular, coordinated inflammatory microenvironment that influences clinical outcomes.

### 2.2. Diabetic kidney disease

Tubulointerstitial inflammation – characterized by infiltration of CD68+ macrophages, CD4+/CD8+ T cells, B cells/plasma cells, mast cells, and eosinophils – is hallmark pathological feature of diabetic kidney disease (DKD) and plays a key role in disease progression. These infiltrates correlate significantly with greater clinical severity, including lower eGFR, higher serum creatinine, and heavier proteinuria, as well as with more advanced histologic damage [37–40]. Notably, CD4+ T cells independently predict the severity of tubular atrophy/interstitial fibrosis (TA/IF) and can mediate mitochondrial injury in tubular epithelial cells [39]. Throughout DKD stages, mast cell numbers and degranulation increase and correlate with markers of tubulointerstitial injury [37]. Mechanistically, hyperglycemia, albuminuria, and dyslipidemia collectively drive a tubulointerstitial inflammatory network *via* activation of the JAK/STAT, CCL2/CCR2, TLR/NF-κB, and NLRP3 pathways.

At the cellular level, interstitial infiltration by CD4+ T cells and eosinophils is associated with more severe disease and worse renal outcomes [39,40]. DKD patients with interstitial eosinophil aggregates are often resistant to glucocorticoids or other immunosuppressants [40]. Mast cell numbers rise with disease progression and contribute to fibrogenesis, supporting their role as indicators of adverse outcomes [37]. CD68+ macrophage infiltration correlates with functional decline and more severe histologic changes, suggesting its utility as a prognostic marker [38]. Since the density of any single cell type shows limited stability for individual-level prediction, more robust prognostic information merges from evaluating the multicellular inflammatory landscape and its integration with the degree of tissue injury.

Multiple studies concur that a higher interstitial inflammatory burden predicts worse outcomes, though its status as an independent predictor varies across cohorts. Okada et al. (in a study of 69 type 2 diabetic patients with heavy proteinuria) identified interstitial lesions – both fibrosis and inflammation – as independent risk factors for renal outcomes [41]. Similarly, Mise et al. in a larger cohort, reported significant effects of IFTA and interstitial inflammation, noting that inflammation in fibrotic areas performed at least as well as, if not better than, inflammation in non-fibrotic areas for prognostic prediction; inflammation restricted to non-fibrotic regions did not outperform that in fibrotic zones [42]. In contrast, An et al. observed that although interstitial inflammation was associated with outcome, this association lost statistical independence after adjustment for baseline log-proteinuria, mean arterial pressure, and eGFR (*p* > 0.05) [43]. Shimizu et al. found more severe interstitial inflammation in patients with heavier albuminuria and/or lower eGFR, highlighting its clinical relevance; however, when incorporated alongside other pathological or clinical covariates, interstitial inflammation did not consistently independently predict renal events, cardiovascular events, or all-cause mortality [44]. Conversely, Hoshino et al. using percentage-based categorizations of interstitial inflammation and adjusting for clinical covariates, found evidence supporting its independent prognostic value [45]. In a related analysis, Shimizu et al. also linked interstitial inflammation to cardiovascular events in type 2 diabetic DKD, suggesting potential systemic implications [44].

The heterogeneity in these findings likely stems from [1]: the lack of uniform indications for renal biopsy in diabetic CKD, leading to cohorts with varying baseline kidney function that may influence effect estimates [2]; divergent grading standards for interstitial inflammation (e.g., total cortex vs. non-fibrotic cortex; percentage categories vs. counts/semi-quantitative scores) [3]; collinearity among pathological variables (particularly with IF/TA and glomerular indices); and [4] differences in the extent of covariate adjustment. Future studies should adopt standardized quantification methods, clearly defined regions, of interest, comprehensive covariate adjustment, and external validation to clarify the incremental prognostic value of interstitial inflammation – and its relationship with fibrotic areas – in DKD.

### 2.3. Lupus nephritis

In lupus nephritis (LN), tubulointerstitial inflammation (TII) is prominent and involves diverse immune cell infiltrates. Interstitial CD4+ T cells, CD8+ T cells, and CD68+ macrophages correlate positively with serum creatinine, glomerulosclerosis proportion, IF/TA, and the chronicity index (CI) [46–48]. Among these, CD4+ T cells show the strongest correlation with CI, while CD8+ T cells also correlate with SLEDAI and proteinuria, suggesting a link to systemic disease activity [46]. Increased macrophage infiltration is associated with renal injury and fibrosis; notably, M2c-like (CD163+) macrophages predominate in the tubulointerstitium [47]. Using spatial transcriptomics and deep learning, Abraham et al. further demonstrated that high B-cell density is more frequent in non-ESRD cases, whereas high CD4-T-cell density – comprising mainly CD8+, γδT, and double-negative (CD4-CD8-) cells – is more common in patients progressing to ESRD; higher CD4-T-cell densities also correlate with greater chronic tubulointerstitial damage [49]. Future studies simultaneously mapping the spatial organization of B cells, T cells, and macrophages may further elucidate these cellular ecosystems.

Mechanistically, immune-complex deposition directly on tubules can activate Fc receptors and complement, triggering TII [50]. Anti-dsDNA antibodies can bind to proximal tubular epithelial cells (PTECs) and induce cytokine secretion that exacerbates inflammation. Mesangial–tubular crosstalk also allows glomerular inflammatory mediators to affect the interstitium [51]. Several non-exclusive pathways may connect glomerular lesions to TII: severe glomerulonephritis can cause tubulointerstitial ischemia–hypoxia; protein leakage due to glomerular damage exposes PTECs to luminal proteins, provoking inflammatory signaling; loss of tolerance initiated at the glomerulus may propagate *via* lymphoid organs and amplify in the interstitium; and severe GN may lead to Bowman’s capsule rupture with spillover inflammation. Importantly, despite mechanistic links, the severity of interstitial nephritis in LN does not always parallel glomerular activity, and each compartment can exhibit distinct immune environment and occur independently [52]. Further research is needed to clarify these compartmental relationships.

Regarding prognosis, Dias et al. found that interstitial CD68+ expression correlates with CKD progression, underscoring the long-term impact of monocyte/macrophage burden even under standard therapy [53]. Chen et al. identified interstitial CD68+ cells as an independent risk factor for adverse outcomes, proposing a cutoff of 340 cells/mm^2^ to predict ESRD within four years (AUC 0.75; sensitivity 87.5%, specificity 61.8%) – though the number of endpoint events was small and macrophage subtypes were not analyzed [48]. Zhang et al. showed significantly worse renal survival in patients with high CD8 + T-cell density; a threshold of >130 cells/mm^2^ independently predicted poorer long-term outcomes [46]. Consistent with spatial analyses, Abraham et al. observed that higher B-cell density correlates with preserved renal function, whereas higher CD4-T-cell density aligns with AKI and progression to ESRD – supporting the concept of distinct *in situ* inflammatory states with divergent prognoses [49].

Historically, the 2003 ISN/RPS classification emphasized glomerular lesions [5]. Subsequent studies established the prognostic importance of tubulointerstitial injury. Yu et al. showed that several tubulointerstitial indices, including interstitial inflammation, are independent predictors of kidney function progression [54]. Hsieh et al. quantified interstitial infiltrates using CD45 staining and graded inflammation by light microscopy after excluding areas near tubular atrophy and fibrosis; they found that the severity of interstitial inflammation – rather than the NIH glomerular activity index – predicted renal failure, and that among components of the NIH chronicity index, only the tubulointerstitial element carried prognostic significance [5]. In contrast, Alsuwaida et al. reported that baseline interstitial inflammation did not predict decline, whereas persistent inflammation on follow-up biopsy strongly predicted ESRD; baseline inflammation did, however, correlated with subsequent fibrosis, supporting an inflammation-fibrosis sequence [55]. Rijnink et al. also advocated systematic recording of interstitial inflammation for risk assessment but emphasized that chronic interstitial damage often provides the strongest independent signal, highlighting the importance of early inflammation control to prevent fibrosis [56]. Wilson et al. found interstitial inflammation to independently predict renal survival after adjusting for demographic and baseline renal parameters [57], while Gomes et al. showed that TII severity remains an independent predictor even after accounting for IF/TA and comorbidities such as hypertension or diabetes [58]. Reflecting this evidence, the 2018 ISN/RPS update introduced a semi-quantitative scoring system that explicitly includes interstitial inflammation (activity index) and IF/TA (chronicity index), elevating the prognostic role of the tubulointerstitium in LN classification [59].

Adding nuance to TII assessment, Duong et al. showed that inflammation within fibrotic areas may be associated with higher ESKD risk; when measured as total cortical interstitial inflammation, the burden correlated with CKD progression and ESKD – suggesting that restricting scoring to non-fibrotic cortex (as in the NIH system) may overlook clinically relevant inflammation in fibrotic zones [60]. Further studies should assess whether whole-cortex quantification offers superior prognostic value in LN.

Finally, treatment-response data are mixed but not necessarily contradictory. The Alsuwaida group found no association between baseline TII severity and likelihood of remission [55]. In contrast, Lan-Ting et al. reported lower response rates in patients with TII [61]; as they compared presence vs. absence (rather than graded severity), their design may be more sensitive to detecting TII-related resistance. Hachiya et al. subsequently showed that higher TII scores correlate with failure to achieve complete remission, supporting the idea that patients with significant interstitial inflammation may require more intensive therapy [62].

### 2.4. Other glomerular diseases

Acute kidney injury (AKI) is a common complication of minimal change disease (MCD), with adults being more susceptible than children. Recent studies indicate that interstitial inflammation is an independent risk factor for AKI in adult patients with MCD [63]; however, its impact on long-term prognosis in this population remains unexplored. In focal segmental glomerulosclerosis (FSGS), interstitial inflammation is a potential risk factor for end-stage kidney disease (ESKD) and is associated with reduced long-term renal survival [64]. Increased expression of FOXP3+ regulatory T cells (Tregs) in FSGS is associated with milder histological lesions [29] and a high FOXP3+/CD4+ T-cell ratio may predict preserved renal function. In a cohort of 16 patients with idiopathic membranous nephropathy (IMN) [65], mesangial – but not glomerular – activated T cells and mononuclear macrophages influenced the initial proteinuric response to corticosteroids but did not predict subsequent relapse. A follow-up study identified interstitial inflammatory infiltrates as an independent risk factor for treatment failure in primary membranous nephropathy [66]. Nonetheless, conclusive evidence regarding the prognostic value of interstitial inflammation in membranous nephropathy is still lacking.

### 2.5. Interstitial inflammation in renal transplantation

In kidney transplant recipients, tubulointerstitial inflammation (TII) is common and closely linked to allograft outcomes. Both the measurement approach and longitudinal dynamics of TII are critical for risk stratification. Mengel et al. reported that persistent inflammatory infiltrates across serial biopsies, are associated with worsened allograft prognosis – even when inflammation is histologically identified in scarred areas [67]. Similarly, the DeKAF study found that assessing inflammation within scarred regions during biopsy evaluation may offer superior prognostic information [68]. Ortiz et al. proposed TII as a major determinant of graft survival, noting that even minimal inflammation on protocol (‘normal’) biopsies is associated with adverse outcomes; recent refinements in Banff inflammation scoring may further improve the stratification of patients at risk of graft loss [69]. Finally, Rodrigo et al. showed that TII is associated with a high risk of CKD stage 5 or death-censored graft loss (DCGL), more than doubling the risk of graft failure and predicting worse outcomes in recipients with recurrent glomerular disease. They recommend that risk assessment in recurrent IgA nephropathy after transplantation should integrate TII, the Oxford classification, and the percentage of crescents to identify candidates most likely to benefit from targeted anti-recurrence strategies [70].

## 3. Pathways in interstitial inflammation

Growing evidence suggests that tubulointerstitial inflammation in glomerular diseases arises from an integrated glomerulo–tubulo–interstitial axis, rather than representing a purely downstream or isolated process. Diverse pathogenic insults initially target the glomerulus, leading to disruption of the filtration barrier and altered glomerular signaling [71].

Glomerular injury transmits to the renal tubules through increased protein filtration, exposure to inflammatory mediators that have been filtered, and metabolic stressors such as high glucose, high lipids, and uric acid. In response, renal tubular epithelial cells activate key inflammatory pathways, including the JAK/STAT, NF-κB, NLRP3 inflammasome, and TLR signaling pathways. The damaged renal tubules then recruit immune cells into the interstitium by releasing chemokines and cytokines, thereby forming a self-sustaining inflammatory cycle [71].

**Figure 1.**
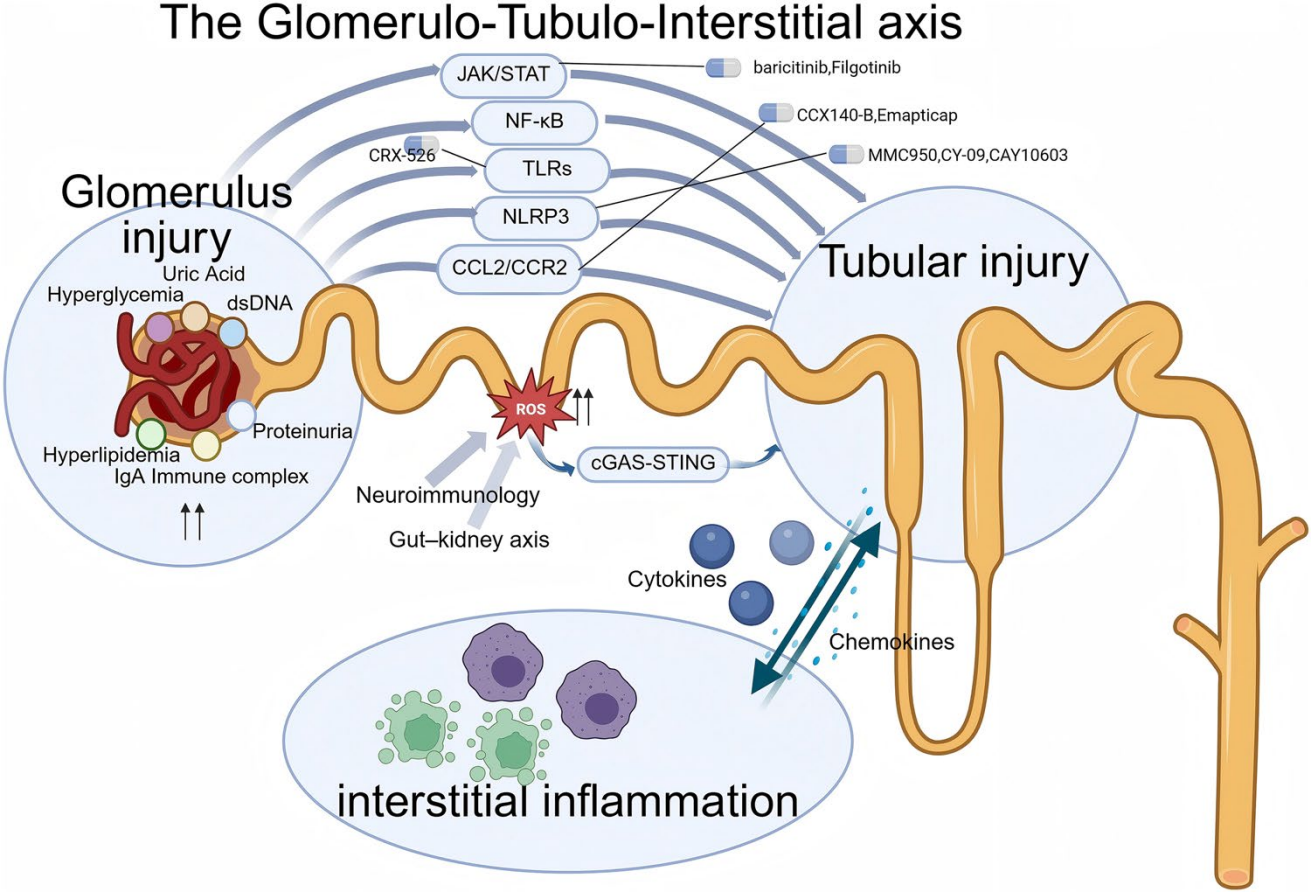
The glomerulo-tubulo-interstitial axis. This figure illustrates the glomerulo–tubulo–interstitial axis, whereby glomerular injury induces tubular stress through proteinuria and metabolic/inflammatory mediators, leading to activation of key inflammatory pathways (JAK/STAT, NF-κB, TLRs, NLRP3, CCL2/CCR2, cGAS–STING). Injured tubules subsequently amplify inflammation by releasing cytokines and chemokines that recruit immune cells into the interstitium, establishing a self-sustaining inflammatory loop and highlighting multiple therapeutic targets and corresponding drugs.(Created in https://BioRender.com)

As illustrated in Figure 1, this glomerulo–tubulo–interstitial axis provides a unifying framework linking glomerular injury to persistent interstitial inflammation across diverse glomerular diseases.

### 3.1. Traditional pathways

#### 3.1.1. JAK/STAT pathway

The JAK/STAT pathway comprises ligand-receptor complex, Janus kinases (JAKs), and signal transducers and activators of transcription (STATs). Upon cytokine binding, receptors dimerize and activate JAKs, which phosphorylate STAT proteins. Phosphorylated STATs form homo- or heterodimers that translocate to the nucleus to regulate gene transcription [72]. In diabetic kidney disease (DKD), JAK/STAT signaling is broadly activated in the renal parenchyma, particularly in the interstitium. Transcriptomic and proteomic analyses reveal that significantly elevated expression of JAK1–3 and STAT1/3 in renal tissues from DKD patients, with JAK2 showing the most marked upregulation [73]. In early diabetic kidney disease, JAK2 expression is predominantly localized to the glomeruli; however, with disease progression, it becomes increasingly evident in the tubulointerstitium, suggesting a potential role in the transition from glomerular injury to interstitial damage [73]. Functional studies further underscore the critical role of the JAK/STAT pathway in regulating renal inflammation. Lu et al. generated transgenic mice with restricted STAT3 activation (Stat3SA/-) and demonstrated that, in a streptozotocin-induced diabetic model, these mice exhibited reduced renal inflammatory cell infiltration, indicating that STAT3 inhibition attenuates chemokine expression and tubulointerstitial immune cell recruitment [74]. The suppressor of cytokine signaling (SOCS) family serves as an endogenous negative regulator of JAK/STAT signaling and is upregulated in diabetic kidney disease [75,76]. Specifically, SOCS1–3 proteins inhibit JAK activation through direct binding. Adenoviral delivery of recombinant SOCS1–3 has been shown to suppress JAK/STAT activity and ameliorate renal tubulointerstitial inflammation [75,76]. Consistently, pharmacological inhibition of JAK1/JAK2 also reduced interstitial inflammation in diabetic rat models [77].

This pathway is also implicated in other glomerulopathies. In IgA nephropathy (IgAN), increased expression of JAK2, phosphorylated STAT1 (pSTAT1), and phosphorylated STAT3 (pSTAT3) has been observed in the tubulointerstitium, where pSTAT signals colocalize with CD3^+^ T cells, suggesting involvement in T cell–driven inflammatory responses [78,79]. Although Tao et al. did not identify a significant correlation between pSTAT expression levels and semiquantitative interstitial inflammation scores, the observed spatial colocalization supports a potential pathogenic role for JAK/STAT signaling in tubulointerstitial immune activation [79]. In focal segmental glomerulosclerosis (FSGS), JAK and STAT proteins are similarly upregulated in the interstitium, with JAK1 colocalizing with CD3^+^ lymphocytes, further implicating this pathway in immune-mediated interstitial inflammation [80]. In lupus nephritis models, pharmacological inhibition of JAK signaling attenuated renal inflammation, as demonstrated by the JAK2 inhibitor AG490 in MRL/lpr mice and the JAK3 inhibitor CP-690550 in NZB/WF1 mice [81,82]. Collectively, these findings indicate that JAK/STAT signaling modulates the inflammatory microenvironment across multiple immune-mediated kidney diseases.

In summary, the JAK/STAT pathway is activated across a spectrum of glomerular diseases, where it promotes pro-inflammatory gene expression and immune cell recruitment within the renal interstitium. Its consistent activation pattern and responsiveness to pharmacological inhibition underscore its relevance as a therapeutic target for renal tubulointerstitial inflammation.

#### 3.1.2. CCL2/CCR axis

CCL2 (monocyte chemoattractant protein-1, MCP-1), is a key CC chemokine that recruits monocytes and macrophages *via* its specific receptor CCR2. The CCL2/CCR2 axis plays a central role in interstitial inflammation and can be activated by metabolic disturbances such as hyperuricemia, hyperglycemia, and dyslipidemia. In a hyperuricemic mouse model, uric acid crystals stimulate tubular epithelial cells to secrete CCL2, promoting macrophages migration and accumulation in the interstitium, where they form M1-dominant inflammatory foci [83,84]. In diabetic nephropathy, high glucose induces CCL2 expression in tubular and endothelial cells, facilitating macrophage chemotaxis and polarization toward a pro-inflammatory M1 phenotype, thereby exacerbating local inflammation [85]. Dyslipidemia also upregulates renal CCL2, attracting macrophages and promoting their conversion into foam cells, which release inflammatory mediators and establish a positive feedback loop between metabolic dysregulation and interstitial inflammation [86].

The CCL2/CCR2 axis is also activated in primary glomerular diseases. MCP-1 mRNA and protein levels are significantly elevated in IgAN patients with moderate to severe interstitial lesions and correlate with monocyte infiltration and tubulointerstitial damage [87]. Lv et al. demonstrated that this axis mediates albumin-induced tubulointerstitial inflammation [88]. CCR2-deficient mice show reduced interstitial inflammation in an Adriamycin nephropathy model, underscoring its role in FSGS pathogenesis [89]. CCL2 inhibition also alleviates interstitial inflammation in MRL/lpr mice with lupus nephritis, confirming its pro-inflammatory function in autoimmune renal injury [90].

In conclusion, the CCL2/CCR2 axis mediates monocyte/macrophage recruitment and activation in the renal interstitium across various glomerular diseases, representing a promising therapeutic target.

#### 3.1.3. NLRP3 inflammasome activation

Inflammasomes are multiprotein complexes central to innate immunity. The NLRP3 inflammasome consists of NLRP3, ASC, and pro-caspase-1, and requires two signals for activation: priming (e.g., NF-κB–mediated transcription of pro-IL-1β and pro-IL-18) and triggering by PAMPs or DAMPs, which promote NLRP3 oligomerization, caspase-1 activation, and maturation of IL-1β and IL-18 [91]. Activators include Advanced glycation end products (AGEs), Reactive Oxygen Species (ROS), Endoplasmic Reticulum (ER) stress, free fatty acids, and uric acid. Key mechanisms involve potassium efflux (e.g., *via* P2X7 receptor activation by ATP), calcium and chloride flux, mitochondrial dysfunction, and ROS-induced TXNIP–NLRP3 binding [92].

NLRP3 activation is most prominent in renal tubular epithelial cells (TECs). In diabetic nephropathy models, NLRP3 is highly activated; genetic knockout or pharmacological inhibition (e.g., with CY-09) reduces interstitial inflammation in STZ-induced or db/db mice [93,94]. In IgAN, NLRP3 is upregulated in TECs, and IgA immune complexes activate the inflammasome in macrophages *via* mitochondrial damage and ROS, leading to IL-1β release. IgA complexes also activate dendritic cells, promoting CD4^+^ T cell responses [19]. NLRP3 inhibition attenuates interstitial inflammation in IgAN models [19], and the NF-κB/NLRP3 pathway is implicated in these responses – compound K, a ginsenoside metabolite, alleviates inflammation *via* this pathway [95].

In SLE, accumulated apoptotic debris provides DAMPs (e.g., ROS, ATP, nucleic acids) that activate NLRP3 [96]. Double-stranded DNA and autoantibodies induce ROS and potassium efflux, amplifying inflammation in monocytes [91]. Impaired clearance of neutrophil extracellular traps (NETs) also activates NLRP3 and IL-18 release, creating a pro-inflammatory loop [91]. In LN patients, NLRP3, ASC, caspase-1, IL-1β, and IL-18 are upregulated in the tubulointerstitium [91]. Although the NLRP3 inhibitor MCC950 reduces inflammation in animal models, its effect on interstitial cells may be limited, suggesting cell-specific differences [97,98]. Albuminuria, acting as a DAMP, also activates NLRP3 *via* mitochondrial ROS, exacerbating interstitial inflammation [99].

In summary, NLRP3 inflammasome activation driven by metabolic, immune, and sterile stimuli promotes pro-inflammatory cytokine production and immune cell infiltration in the renal interstitium, representing a key pathological mechanism in glomerular diseases.

#### 3.1.4. Toll-like receptor signaling

Toll-like receptors (TLRs) are pattern recognition receptors (PRRs) that recognize PAMPs and DAMPs, initiating tubulointerstitial inflammation mainly *via* NF-κB activation and pro-inflammatory gene expression. TLRs can also synergize with other PRRs (e.g., NLRP3) to amplify inflammatory cascades [100].

In metabolic kidney diseases, endogenous ligands such as uric acid crystals, non-crystalline uric acid, and AGEs bind TLRs, activating NF-κB and inducing CCL2 and ICAM-1 expression, which promotes monocyte recruitment and adhesion [101–103]. Lipid ligands can also activate CD36/TLR complexes and Na^+^/K^+^-ATPase, triggering NF-κB in TECs and macrophages [104,105].

In DKD, TLR4 expression is increased in tubules and correlates with interstitial macrophage infiltration. High glucose upregulates TLR4 *via* PKC, activating NF-κB and inducing IL-6 and CCL2 [106]. TLR4^-^/^-^ diabetic mice show reduced NF-κB activation and interstitial inflammation [106]. In chronic diabetic inflammation, however, TLR2 may be more critical: HMGB1 activates NF-κB through both TLR2 and TLR4, but after prolonged high-glucose exposure, only TLR2 remains upregulated, suggesting dominance of the HMGB1–TLR2–NF-κB axis in sustained inflammation [107]. Albumin overload induces TLR4-dependent inflammation by stimulating HSP70 release from TECs; HSP70 then activates TLR4, and blocking this pathway mitigates albumin-induced inflammation [108].

In LN, TLR expression is linked to interstitial inflammation. TLR9 is upregulated in tubules of SLE patients and MRL/lpr mice, while TLR3, TLR7, and TLR9 are widespread in LN tubulointerstitium, with TLR7 associated with chronicity and TLR9 with activity indices and systemic scores [109,110]. In MRL/lpr mice, Nrf2 inhibits inflammation *via* the Nrf2/HMGB1/TLR4/NF-κB axis [111].

In conclusion, TLR signaling initiates and sustains renal interstitial inflammation in metabolic and immune-mediated diseases through NF-κB activation, making TLRs potential therapeutic targets for controlling interstitial inflammation.

**Figure 2.**
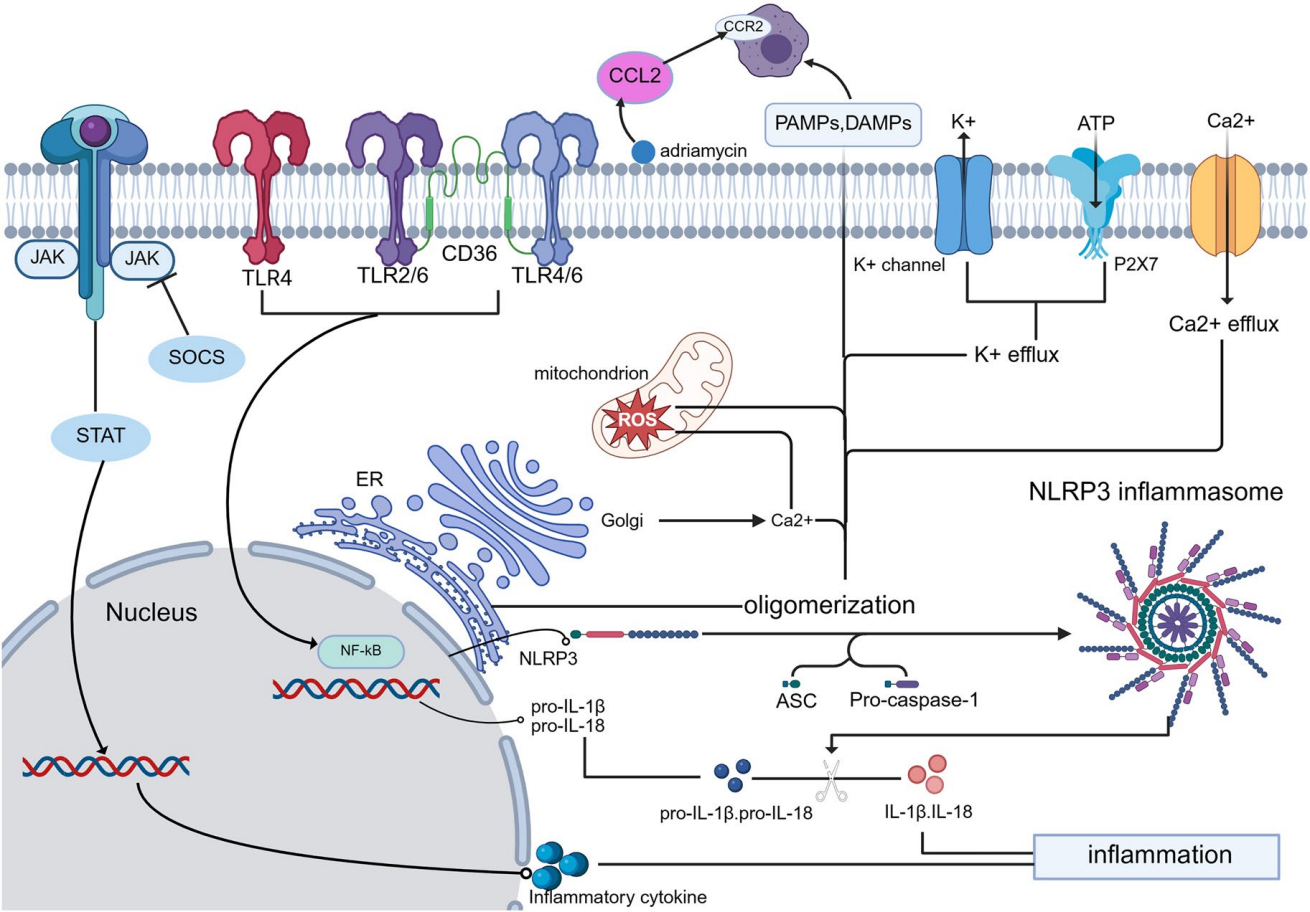
The classical traditional pathway in interstitial inflammation. This figure depicts classical traditional inflammation pathway in Interstitial inflammatory, mainly including the JAK/STAT signaling pathway, CCL2/CCR axis, NLRP3 inflammasome activation, and Toll-like receptor signaling. (Created in https://BioRender.com)

The pathways associated with interstitial inflammation mentioned above are succinctly summarized in Figure 2.

### 3.2. Emerging pathways

#### 3.2.1. Mitochondrial innate immunity

The cyclic GMP–AMP synthase–stimulator of interferon genes (cGAS–STING) pathway is a central component of the innate immune system that senses cytosolic double-stranded DNA. Under physiological conditions, mitochondrial DNA (mtDNA) is strictly confined to the mitochondrial matrix. However, disruption of mitochondrial integrity leads to mtDNA fragmentation and cytosolic leakage, where it is recognized by cGAS, triggering the production of cyclic GMP–AMP (cGAMP). cGAMP subsequently activates STING at the endoplasmic reticulum, initiating downstream TBK1–IRF3 and NF-κB signaling cascades and inducing the expression of type I interferons and proinflammatory cytokines [112].

In the kidney, this mitochondrial innate immune axis is increasingly recognized as an upstream driver of tubular injury and tubulointerstitial inflammation. Kidney exhibits one of the highest mitochondrial densities and oxygen consumption rates in the human body. Tubular epithelial cells rely heavily on mitochondrial oxidative phosphorylation to meet the high energy demands of solute reabsorption, rendering them particularly vulnerable to mitochondrial stress. Mitochondrial dysfunction results in excessive reactive oxygen species (ROS) production, redox imbalance, and oxidative stress, thereby promoting inflammation, fibrosis, and progressive renal injury. Concurrently, damaged mitochondria release mtDNA and other damage-associated molecular patterns (DAMPs), which activate the cGAS–STING pathway and amplify inflammatory signaling and immune cell recruitment within the interstitium, accelerating disease progression [113].

Mitochondrial transcription factor A (TFAM) is essential for mtDNA maintenance and stability. Reduced expression of TFAM and other mitochondrial proteins has been documented in patients with chronic kidney disease and in experimental models of renal fibrosis, accompanied by elevated inflammatory cytokine levels [114]. In tubular-specific TFAM knockout mice, impaired mtDNA packaging leads to cytosolic mtDNA accumulation and spontaneous activation of the cGAS–STING pathway, resulting in tubular injury, interstitial immune cell infiltration, and progressive fibrosis. Genetic deletion or pharmacological inhibition of STING significantly attenuated tubulointerstitial inflammation in this context [114]. Similarly, in an angiotensin II–induced hypertensive nephropathy model, pathological mtDNA release from tubular epithelial cells activated STING-dependent inflammatory cascades, promoting macrophage recruitment and fibrotic responses. Notably, inhibition of acyl-CoA synthetase long-chain family member 4 (ACSL4) exerted comparable anti-inflammatory effects by suppressing mtDNA-driven STING activation, highlighting the STING–ACSL4 axis as a potential therapeutic target in hypertension-associated chronic kidney disease [115]. Beyond tubular injury, aberrant STING signaling has also been implicated in immune-mediated and metabolic glomerular diseases. In lupus nephritis models, increased STING expression was observed, and STING inhibition effectively reduced interstitial inflammation, underscoring the pathogenic role of the STING–TBK1–NF-κB axis in disease progression [116]. In diabetic nephropathy, Mitrofanova et al. reported elevated STING expression in podocytes, which contributed to podocyte injury and proteinuria, although STING activation was not detected in the tubulointerstitium and was not associated with immune cell infiltration [117]. In contrast, Yang et al. demonstrated that genetic deletion of STING alleviated podocyte injury and suppressed tubulointerstitial inflammation by attenuating NLRP3 inflammasome activation [118] suggesting that glomerular STING activation may indirectly drive interstitial inflammation through proteinuria-mediated mechanisms.

#### 3.2.2. Senescence-associated secretory phenotype

Cellular senescence is an irreversible state of cell cycle arrest accompanied by profound alterations in chromatin organization, metabolism, and paracrine signaling. Although senescent cells permanently exit the cell cycle, they remain metabolically active and secrete a broad array of bioactive factors, including cytokines, chemokines, growth factors, and proteases. This proinflammatory secretory program, termed the senescence-associated secretory phenotype (SASP), exerts potent effects on the surrounding microenvironment and neighboring cells [119]. Senescence can be induced by multiple stressors, including oxidative stress, chronic inflammation, hyperglycemia, genotoxic insults (e.g., carcinogens, chemicals, and radiation), oncogene activation, tumor suppressor gene loss, mitochondrial dysfunction, telomere attrition, endoplasmic reticulum stress, and uremia [120].

In the kidney, tubular epithelial cells (TECs) are considered a primary site of renal aging. Owing to their high metabolic demands and sustained exposure to ischemia, toxins, and metabolic overload, TECs are particularly susceptible to cellular injury and senescence. Accumulating evidence indicates that cellular senescence increases vulnerability to nephropathy, whereas chronic kidney disease itself promotes immune senescence, thereby establishing a feed-forward loop that amplifies renal inflammation and disease progression. These interactions have been comprehensively reviewed elsewhere [121,122].

Senescence of tubular epithelial cells has been documented across multiple renal disorders, including diabetic nephropathy, IgA nephropathy, ischemic nephropathy, and chronic interstitial nephritis [123]. Increased expression of the senescence marker p16 has been observed in the renal interstitium of patients with glomerular diseases and was positively correlated with the severity of interstitial inflammation, suggesting that disease-associated senescence of interstitial cells contributes to inflammatory activation [124]. Consistently, elevated expression of senescence markers such as p16 and p21 has been detected in tubular epithelial cells from patients with IgA nephropathy, with levels correlating positively with tubulointerstitial inflammation [125]. Both *in vitro* and *in vivo* studies further demonstrate that hyperglycemia promotes TEC senescence and SASP production, thereby enhancing inflammatory cell recruitment and extracellular matrix accumulation within the tubulointerstitium [126].

In summary, tubular epithelial cell senescence functions as an upstream ‘inflammatory senescence’ module within the peritubular interstitium. Persistently senescent TECs sustain chronic SASP signaling, drive immune cell recruitment and phenotypic reprogramming, and promote interstitial inflammation and fibrosis. Accordingly, therapeutic strategies aimed at eliminating senescent tubular cells or modulating SASP signaling may represent promising approaches for mitigating tubulointerstitial inflammation and fibrotic progression in glomerular diseases and chronic kidney disease.

#### 3.2.3. Neuro-immune system

The autonomic nervous system consists of sympathetic and parasympathetic branches and plays a central role in maintaining physiological homeostasis. Autonomic dysfunction is highly prevalent in patients with chronic kidney disease (CKD) and is typically characterized by sympathetic overactivity and reduced parasympathetic tone [127]. This imbalance is closely associated with hypertension, atherosclerosis, and progressive structural and functional renal impairment. These processes reinforce one another, forming a vicious cycle that accelerates CKD onset and progression.

The kidney is a densely innervated organ. The renal plexus is predominantly composed of visceral sympathetic fibers, with norepinephrine serving as the principal neurotransmitter. Renal afferent sensory nerves, primarily distributed around the renal pelvis, transmit mechanical and chemical signals from the kidney to the central nervous system. In contrast, efferent sympathetic nerves course along the renal vasculature and regulate renal blood flow, glomerular filtration rate, sodium and water reabsorption, and the release of renin and prostaglandins. In parallel, the vagus nerve–mediated brain–kidney axis participates in the regulation of renal vascular tone and sodium excretion [128].

As reviewed by Zoccali et al. CKD is consistently associated with heightened sympathetic activity and impaired parasympathetic regulation [129]. These alterations are driven by persistent activation of renal afferent nerves in diseased kidneys, excessive stimulation of the renin–angiotensin system (RAS), and accumulation of uremic solutes such as urea and adenosine. Together, these signals activate central sympathetic nuclei, further augmenting renal sympathetic outflow and establishing a self-perpetuating feedback loop between the injured kidney and the central nervous system.

Beyond hemodynamic regulation, the autonomic nervous system plays a critical role in modulating renal inflammation. Norepinephrine and other catecholamines released from sympathetic nerve terminals act on adrenergic receptors expressed by renal tubular epithelial cells, interstitial macrophages, and T lymphocytes. This stimulation enhances reactive oxygen species production and activates pro-inflammatory signaling pathways, including NF-κB and JAK/STAT, leading to increased expression of cytokines and chemokines. These mediators promote immune cell adhesion, infiltration, and persistence within the tubulointerstitium, thereby driving chronic inflammation and fibrotic remodeling. In contrast, parasympathetic signaling through the vagus nerve activates α7 nicotinic acetylcholine receptors on immune cells *via* the cholinergic anti-inflammatory pathway, suppressing pro-inflammatory cytokine production, limiting leukocyte recruitment, and exerting a protective effect against interstitial inflammation [129]. Accordingly, sympathetic hyperactivity coupled with vagal dysfunction in CKD not only exacerbates hemodynamic and metabolic disturbances but also sustains tubulointerstitial inflammation through dysregulated neuroimmune interactions, accelerating fibrotic progression toward end-stage renal disease.

Experimental studies provide functional support for this ‘neuro–immune–renal interstitial’ axis. Renal autonomic denervation has been shown to reduce renal norepinephrine levels, diminish immune cell infiltration, and downregulate inflammatory cytokine expression, thereby alleviating tubulointerstitial inflammation and fibrosis in models of glomerular disease and renal ischemia. Conversely, enhancement of vagal nerve activity or pharmacological activation of α7 nicotinic acetylcholine receptors suppresses interstitial inflammation and tissue injury through cholinergic anti-inflammatory mechanisms [130–132]. Notably, angiotensin-converting enzyme inhibitors (ACEIs) and angiotensin receptor blockers (ARBs), which remain first-line therapies for glomerular diseases, also attenuate tubulointerstitial inflammation and fibrosis by inhibiting RAS-driven sympathetic activation and angiotensin II–mediated pro-inflammatory signaling. These observations underscore the clinical relevance of neurohumoral–immune interactions in regulating renal interstitial inflammation [6,133].

#### 3.2.4. Gut–kidney–interstitium axis

The gut–kidney axis describes the bidirectional interactions between the gut microbiota and renal function that are essential for maintaining systemic metabolic and immune homeostasis. Under physiological conditions, the gut microbiota supports nutrient absorption, metabolite production, and immune tolerance [134]. In the context of kidney health, its principal functions involve modulation of inflammation, uremic toxin burden, and metabolic balance. The gut microbiota is composed of diverse bacterial, fungal, and viral communities, with Firmicutes and Bacteroidetes representing the dominant phyla. In CKD, intestinal dysbiosis is commonly observed and is characterized by a reduction in beneficial taxa such as *Lactobacillus*, *Prevotella*, and *Bifidobacterium*, alongside an expansion of potentially pathogenic bacteria, including *Proteobacteria* and *Enterococcus* [135].

The mechanisms underlying CKD-associated dysbiosis are multifactorial and may involve uremic toxin accumulation, metabolic acidosis, oral iron and phosphate binder use, impaired intestinal barrier integrity, reduced dietary fiber intake, and constipation. These changes alter microbial metabolite profiles, leading to increased production of protein-bound uremic toxins – such as indoxyl sulfate (IS), p-cresol sulfate (PCS), and trimethylamine N-oxide (TMAO) – as well as disturbances in short-chain fatty acids, advanced glycation end products, and ketone bodies. Collectively, these alterations promote oxidative stress and inflammatory signaling within the kidney, disrupt the tubulointerstitial microenvironment, and accelerate disease progression [135].

IS and PCS are protein-bound uremic toxins derived from bacterial metabolism of dietary tryptophan and tyrosine, respectively. IS induces reactive oxygen species generation in renal tubular epithelial cells, leading to oxidative stress and activation of transcriptional regulators such as NF-κB and p53. This process upregulates chemokine expression and promotes inflammatory cell infiltration within the renal interstitium [136]. Similarly, PCS stimulates interstitial monocyte and macrophage accumulation and increases the expression of pro-fibrotic mediators, including IL-6 and TGF-β, thereby contributing to renal fibrosis [137]. Both IS and PCS have been shown to induce hypermethylation of the *Klotho* gene, suppressing its expression and diminishing its renoprotective effects, ultimately accelerating renal functional decline [138]. Consistent with these findings, animal studies demonstrate that IS and PCS exacerbate tubulointerstitial injury and fibrosis in CKD models [139].

TMAO is a gut-derived metabolite generated through microbial metabolism of choline, carnitine, and phosphatidylcholine. Within the renal microenvironment, TMAO promotes inflammation through multiple mechanisms. It enhances expression of the scavenger receptor CD36 in infiltrating interstitial macrophages, facilitating foam cell formation and downstream inflammatory cascades. TMAO also induces potassium efflux in tubular epithelial cells, activating the NLRP3 inflammasome and caspase-1–dependent IL-1β maturation. In addition, TMAO amplifies oxidative stress signaling, further aggravating inflammatory responses [140]. Experimental studies demonstrate that dietary or exogenous TMAO supplementation worsens renal dysfunction, tubulointerstitial inflammation, and fibrosis, whereas inhibition of microbial TMAO production or systemic TMAO reduction attenuates interstitial injury and improves renal outcomes [141,142].

In addition to microbial metabolites, circulating endotoxins such as lipopolysaccharide activate Toll-like receptors on renal cells, triggering pro-inflammatory and oxidative stress pathways that directly damage the tubulointerstitium. Conversely, short-chain fatty acids produced through microbial fermentation of dietary carbohydrates exert anti-inflammatory effects and help preserve intestinal barrier integrity. Reduced short-chain fatty acid availability compromises barrier function, increases intestinal permeability, and promotes endotoxemia, thereby amplifying renal inflammation and oxidative stress [143].

## 4. Diagnostic strategies

The diagnosis of renal interstitial inflammation primarily relies on renal biopsy and pathological analysis. Clinicians begin with a history and physical examination, assessing proteinuria, serum creatinine, and estimated glomerular filtration rate (eGFR) to determine whether a biopsy is indicated. Biopsy specimens are evaluated for interstitial inflammatory infiltrates using immunohistochemistry, Masson’s trichrome, or periodic acid–Schiff (PAS) staining. Pathological evaluation criteria include: (i) interstitial cellular infiltration and its composition (monocytes/macrophages, T and B lymphocytes); (ii) tubular and interstitial injury – such as tubular atrophy, interstitial fibrosis, and edema; and (iii) immunohistochemical quantification of CD68^+^, CD3^+^, CD20^+^, and IL-1β^+^cell [144]. Due to the invasive nature of renal biopsy, noninvasive biomarkers are under active investigation. In recent years, a range of biomarkers derived from blood and urine, together with advanced imaging modalities, have emerged as promising tools for predicting tubulointerstitial injury (Table 1). Owing to the accessibility of urine, the identification of sensitive and specific urinary biomarkers is particularly valuable for the noninvasive quantification of tubulointerstitial damage.

**Table 1.** Potential predictive markers related to tubulointerstitial inflammation.

Type	Marker/tracer	Diagnostic performance	Advantages/observed association	Limitation	Verification status
Urine					
	MCP-1 [188–192]	NR	Classic chemokine, directly reflecting macrophage recruitment; Repeatedly verified in various kidney diseases;	Affected by proteinuria and eGFR	Validation
	NGAL [191,193]	NR	Sensitive to renal tubular damage; elevated in the context of acute or active kidney injury.	Mainly reflects tubular injury rather than inflammatory cell infiltration, influenced by factors such as AKI, infection, and proteinuria.	Validation
	NAP[194,195]	AUC = 0.846 sensitivity 96.7% specificity 76.0%	Has good diagnostic performance for active lesions in LN; Related to an active inflammatory state.	Strong disease specificity; Insufficient validation.	Validation
	VDBP [196]	NR	Proximal tubule reabsorption function is highly sensitive to abnormalities.In Less affected by proteinuria.	Mainly reflects tubular reabsorption impairment rather than inflammatory cell infiltration; Insufficient validation.	Discovery
	CD163 [11,197]	NR	In LN, they are associated with active lesions and inflammatory burden, and do not rely entirely on proteinuria.	The queue size is limited, and there is a lack of cross-disease validation. The detection method has not been standardized.	Discovery
	AGT [198]	AUC= 0.664	Reflects activation of intrarenal RAAS and is related to the degree of chronic kidney injury.	Limited direct association with TII.	Discovery
	HO-1 [199]	NR	Inflammatory/Oxidative Stress-Induced Molecules; elevated in Inflammatory Kidney Diseases	Insufficient specificity; Mostly discovery-phase research	Discovery
	miRNA-192 [200]	NR	In IgAN, related to TII	The boundaries between inflammation and fibrosis are unclear; Insufficient validation	Discovery
	miRNA-19b-3p [201]	NR	It has been found to be associated with TII in DN patients and FSGS animal models.	Mostly correlational studies Lack direct pathological correlation with specific inflammatory cell infiltration;	Discovery
	CCL2 mRNA [88]	AUC= 0.828	Directly reflects the CCL2-CCR2 axis, which is highly associated with monocyte/macrophage recruitment	mRNA detection has not been standardized and has poor clinical accessibility.	Discovery
	CCL21 mRNA [202]	NR	It has been found to be associated with TII in DN patients.	There is limited direct pathological comparison with TII. Clinical validation is scarce and still in the exploratory stage.	Discovery
Blood					
	KIM-1 [188,203,204]	NR	Highly sensitive to proximal tubule injury;Stable in AKI and some interstitial diseases	Primarily reflects tubular injury; Limited association with inflammatory TII; Not suitable for evaluating inflammatory cell infiltration.	Validation
	miRNA-21-5p [205]	NR	Related to the inflammation-repair-fibrosis pathway. In IgAN, related to TII.	It is more strongly related to IF/TA and fibrosis than to inflammatory cell infiltration; Insufficient specificity for inflammation.	Discovery
	miRNA-192 [200]	NR	In IgAN, related to TII.	The boundaries between inflammation and fibrosis are unclear;	Discovery
MRI					
	T1	NR	It is more inclined toward chronic damage	Inflammation and ischemia are difficult to distinguish	Discovery
	BOLD-MRI	NR	Reflects the oxygenation status of the organization and is related to inflammatory-metabolic-microcirculatory changes	Does not directly reflect inflammatory cell infiltration Prone to influence from hemoglobin and hemodynamics; Inflammation and ischemia are difficult to distinguish	Discovery
	DWI/ADC	NR	No contrast agent, suitable for CKD patients. Sensitive to renal parenchymal cell density and interstitial edema.	Limited ability to distinguish between inflammation and fibrosis	Discovery
US					
	Vmean	NR	Noninvasive, contrast-free, and bedside repeatable. Preliminary studies suggest: They may be more sensitive in distinguishing inflammation and pure fibrosis.	The biological significance of the adhesive parameter remains only partially understood; Insufficient technical standardization.	Discovery
PET					
	FAP-PET ([^68^Ga]Ga-FAPI)	NR	Visualization of stromal activation response; It has been shown to be highly sensitive to fibrotic/repair lesions in various organs	High cost and limited accessibility; Limited ability to distinguish between inflammation and fibrosis; Risk of kidney damage.	Discovery
	[^18^F] AlF-NOTA-FAPI-04	NR	Visualization of stromal activation response; It has been shown to be highly sensitive to fibrotic/repair lesions in various organs	High cost and limited accessibility; Limited ability to distinguish between inflammation and fibrosis	Discovery

*Abbreviation:* NR, not reported.

Compared with urinary markers, imaging techniques provide continuous spatial information across the entire kidney. Magnetic resonance imaging (MRI), ultrasound (US), and molecular positron emission tomography (PET) enable the evaluation of tubulointerstitial inflammation and fibrosis at microstructural, mechanical, and cellular–molecular levels, respectively. Diffusion-weighted imaging (DWI) and its derivative intravoxel incoherent motion (IVIM) models indirectly reflect changes in interstitial cell density and extracellular space by quantifying water diffusion restriction. Inflammatory cell infiltration and interstitial expansion increase diffusion restriction, leading to a reduction in the apparent diffusion coefficient (ADC) [145]. T1 mapping generates parametric maps in which each voxel represents tissue-specific T1 relaxation times and is more indicative of chronic injury, although its ability to distinguish inflammation from fibrosis remains limited [146]. Blood oxygen level–dependent (BOLD) MRI assesses renal tissue oxygenation but is influenced by hemodynamic factors and anemia [147]. Collectively, these MRI parameters correlate with interstitial inflammation, fibrosis severity, and estimated glomerular filtration rate (eGFR). However, ADC and T1 mapping demonstrate greater sensitivity for fibrosis than for active inflammation [145–148].

Ultrasound-based techniques further enable mechanical characterization of renal tissue. Shear wave elastography allows quantitative assessment of tissue elasticity and has been associated with interstitial fibrosis and declining renal function. Using the strain grid finite difference (SGFD) method to simulate heterogeneous shear wave propagation in renal cortical tissue, one study demonstrated that renal allografts with inflammation exhibited distinct spatial mechanical signatures compared with normal tissue, characterized by increased elasticity and viscosity, whereas fibrotic tissue showed minimal changes [149]. In a larger cohort study of 332 patients with CKD and 190 healthy controls, ultrasound viscoelastic imaging revealed that the viscosity parameter (Vmean) correlated with both interstitial inflammation and fibrosis [150].

Molecular imaging, particularly PET, provides a novel approach for visualizing inflammatory cell populations *in vivo*. PET imaging with targeted radiotracers enables noninvasive, quantitative assessment of specific molecular and cellular processes. Several macrophage-associated targets, including translocator protein (TSPO), CD206, CD163, and P2X7 receptor, are under investigation, although applications in renal disease remain limited. A recent study pioneered the use of ^11^C-CPPC PET targeting colony-stimulating factor 1 receptor (CSF1R) to visualize predominantly M2-type macrophages in an ischemia–reperfusion acute kidney injury model, demonstrating a close correlation between PET signal intensity and activated macrophage accumulation [151]. In parallel, fibroblast activation protein (FAP)–targeted PET imaging ([^68^Ga]Ga-FAPI) has shown increased uptake in regions of interstitial fibrosis [152]. More recently, a novel tracer, [^18^F]AlF-NOTA-FAPI-04, was evaluated in IgA nephropathy, where renal parenchymal uptake correlated positively with disease severity, as assessed by the Oxford classification, and with the extent of interstitial inflammation and fibrosis [153]. Compared with ^68^Ga-labeled tracers, [^18^F]AlF-NOTA-FAPI-04 offers higher radiolabeling yield, greater specific activity, and a longer half-life, facilitating large-scale production and potentially improving image quality. Similar findings have been reported across a broader spectrum of renal diseases [154], supporting the potential of FAPI PET/CT as a clinically applicable, noninvasive modality for comprehensive assessment of tubulointerstitial inflammation.

## 5. AI-augmented pathology and spatial transcriptomics: mapping the interstitial immune ecosystem

Conventional renal biopsy assessment of tubulointerstitial inflammation relies predominantly on semi-quantitative scoring systems. Interobserver variability, sampling bias, and limited discrimination between patchy inflammation and fibrosis-associated inflammatory infiltrates hinder the translation of interstitial inflammation into reproducible, continuous endpoints suitable for clinical trials. Moreover, heterogeneity in scoring systems, sampling regions, and covariate adjustment strategies contributes to inconsistent prognostic value across different glomerular diseases, limiting the utility of interstitial inflammation for risk stratification and precision therapy.

Advances in digital pathology and artificial intelligence have enabled high-throughput whole-slide image (WSI) acquisition, providing a foundation for objective feature quantification and the discovery of subvisual histopathological patterns. Current machine learning approaches in computational pathology are broadly classified as strongly supervised, weakly supervised, or unsupervised. Strongly supervised methods depend on pixel- or region-level annotations and are well suited for structural segmentation and cell identification but are labor-intensive and difficult to scale. Unsupervised approaches facilitate phenotypic clustering and feature pretraining. Weakly supervised methods, which require only slide- or case-level labels, are particularly compatible with renal pathology, given limited biopsy tissue, lesion heterogeneity, and the impracticality of exhaustive annotations [155].

Within this paradigm, CLAM and SlideGraph + exemplify attention-based multi-instance learning (MIL) and graph-structured WSI modeling, respectively. CLAM treats WSIs as collections of image tiles and, using slide-level labels alone, identifies regions most relevant to clinical outcomes while generating interpretable attention heatmaps for lesion localization and continuous morphological quantification [156]. In contrast, SlideGraph + represents tissue microregions as nodes connected by edges defined by spatial proximity or morphological similarity, enabling graph neural networks to model higher-order tissue organization and cellular interactions. While MIL approaches emphasize identifying critical regions, graph-based models focus on spatial topology and coordinated tissue architecture, integrating both cellular and contextual information [157].

Although originally developed outside nephropathology, these frameworks have been widely adopted in renal digital pathology. As summarized by Hölscher et al. AI-driven renal pathology now spans the full analytical pipeline from compartment segmentation and lesion localization to continuous quantification, spatial coupling analysis, and outcome prediction [155]. Specifically, renal compartments such as glomeruli, tubules, interstitium, and vessels are first detected and segmented, followed by quantification of pathological features including interstitial fibrosis and tubular atrophy (IFTA), interstitial inflammation, tubular injury, and vascular lesions. Subsequent modeling of spatial co-localization patterns between inflammation and fibrosis further enhances whole-slide–based prognostic prediction.

Building on this foundation, AI-enhanced pathology provides a computational framework for quantifying previously elusive concepts such as ‘immune–fibrotic niches’. MIL-based models can automatically identify microregions characterized by coexisting inflammatory cell enrichment, tubular injury, and matrix expansion, integrating these local patterns into continuous, section-level risk phenotypes. Graph neural network models further capture spatial adjacency and morphological coupling between tissue microdomains, enabling refined characterization of the patchy co-localization of inflammation and fibrosis within the renal interstitium. This spatially informed analysis moves beyond simple density measurements, embedding immune infiltration within high-dimensional representations of tissue organization and improving outcome prediction across heterogeneous glomerular diseases.

Importantly, the spatial information derived from digital pathology enables integration with transcriptomic and proteomic data. Recent studies demonstrate that combining bulk or single-cell RNA sequencing, spatial transcriptomics, proteomics, and digital pathology can reconstruct tissue-level molecular–morphological maps of kidney disease, linking histological phenotypes to their underlying molecular programs [13]. Transcriptomic data elucidate inflammatory and fibrotic states of immune cells, tubular epithelial cells, and fibroblasts, while proteomics captures downstream effector molecules. Digital pathology provides the spatial context in which these molecular states are organized. Through multimodal alignment and joint modeling, shared and disease-specific patterns of tubulointerstitial inflammation can be systematically characterized across glomerular diseases, enabling construction of a cross-disease tubulointerstitial inflammation atlas linked to prognostic risk and therapeutic targets.

Within this framework, AI evolves from a tool for automated image interpretation into an integrative platform connecting tissue morphology, spatial molecular features, and clinical outcomes. By jointly modeling immune–fibrotic patterns from WSIs with transcriptomic and proteomic signatures, this approach enables more precise risk stratification, patient subtyping, and the development of targeted therapeutic strategies in precision nephrology.

## 6. Therapeutic targets

Although conventional treatments have advanced the management of underlying kidney diseases, targeted therapies for interstitial inflammation – a common pathological feature across nephropathies – remain underdeveloped. Recently, interventions targeting key inflammatory pathways such as JAK/STAT, NLRP3, CCL2/CCR2, and TLRs have entered clinical trials (Table 2). The following section evaluates the translational potential and current limitations of these approaches.

**Table 2.** Current clinical trials of novel targeted inflammatory signaling pathway therapy for glomerular diseases.

Mechanism of action	Drug	Outcomes/Research endpoint	Main side effects/safety end point	trial population	phase
JAK1 and JAK2	baricitinib	UACR and inflammatory markers reduced	Well tolerated	Type 2 diabetic nephropathy (eGFR 25–70 mL/min/1.73 m² and UACR 300–5,000 mg/g)	Phase II clinical trial (NCT01683409)
		The primary efficacy end point is percent change in UACR from baseline to end of month 6	incidence of a clinically significant decrease in hemoglobin of ≥ 1 g/dL	Proteinuric APOL1-Related FSGS and Non-diabetic APOL1-related hypertensive nephropathy	Phase II clinical trial (Still in progress) (NCT03615235)
JAK1	Filgotinib	24-h urinary protein decreased	neutropenia and bronchitis	LMN (Renal biopsy confirmation time ≤3 years, eGFR ≥40 mL/min/1.73 m², urinary protein ≥1.5 g/day)	Phase II clinical trial (NCT03285711)
CCL2	CCX140-B	UACR reduced by 10–15% after one year versus placebo	Well tolerated	Type 2 diabetes with proteinuria (UACR 100–3000 mg/g; eGFR ≥ 25 mL/min/1.73 m^2^)	Phase II clinical trial (NCT01447147)
		UPCR has not shown a significant decrease	Well tolerated	Identified podocyte mutations as well as biopsy-proven primary FSGS	Phase I clinical trial (NCT03536754)
	Emapticap	UACR reached its lowest value (−26%) after 2-months’ discontinuing the medication	Well tolerated	Albuminuric type 2 diabetics	Phase IIa clinical trial (NCT01547897)

JAK/STAT inhibitors are widely used in immune-mediated diseases such as psoriasis, ankylosing spondylitis, rheumatoid arthritis, and inflammatory bowel disease. In a phase 2 randomized controlled trial (RCT), baricitinib was tested in patients with type 2 diabetes–associated chronic kidney disease, eGFR 25–70 mL/min/1.73 m^2^, and urine albumin-to-creatinine ratio (UACR) 300–5,000 mg/g. After 24 weeks, baricitinib significantly reduced UACR and inflammatory markers. Safety data revealed a dose-dependent risk of anemia, particularly with the 4 mg/day dose, though no unexpected adverse events occurred. The trial was limited by moderate sample size, limited statistical power, and short duration. Future studies should include larger cohorts and patients with lower degrees of albuminuria to improve generalizability [158]. Baricitinib is currently under investigation in a phase 2 trial for apolipoprotein L1 (apoL1)-related FSGS and hypertensive nephropathy [159]. Another open-label phase 2 study evaluated filgotinib (200 mg/day) in nine patients with lupus membranous nephropathy (LMN), eGFR ≥40 mL/min/1.73 m^2^, and proteinuria ≥1.5 g/day. At week 16, 24-h urinary protein decreased by up to 50.7%. Common adverse events included neutropenia and bronchitis. The lack of a control group and small sample size preclude definitive conclusions regarding efficacy and safety, warranting larger controlled trials [160].

The CCR2 antagonist CCX140-B reduced proteinuria and podocyte injury and improved glycemic control in animal models [161]. In a trial of 332 DKD patients on renin-angiotensin system (RAS) inhibition with UACR 100–3,000 mg/g and eGFR ≥25 mL/min/1.73 m^2^, CCX140-B reduced UACR by 10–15% after one year versus placebo [162]. In a high-baseline UACR subgroup (>800 mg/g), proteinuria decreased by 28% and eGFR decline slowed. The anti-proteinuric effect was sustained for at least 1 month, without affecting blood pressure or weight. The 5 mg/day dose was more effective than 10 mg/day, and fasting glucose improved. Compensatory increases in circulating CCL2 were noted, especially at higher doses [162]. However, in the LUMINA-1 trial involving 46 patients with primary FSGS, CCX140-B (15 mg BID) did not significantly reduce UPCR compared to placebo at 12 weeks. No serious adverse events were reported, but development for FSGS was discontinued due to limited efficacy [163]. Another CCL2 inhibitor, emapticap pegol (NOX-E36), improved proteinuria in a T2DM model by reducing macrophage infiltration and oxidative stress while protecting podocytes [164]. In a phase 2a RCT involving 75 T2DM patients with residual proteinuria despite RAS blockade, emapticap pegol given twice weekly subcutaneously for 12 weeks led to a non-significant UACR reduction trend (–26%, *p* = 0.064) that was maximal 2 months post-treatment. After excluding confounders, UACR reduction reached 39%. No hemodynamic changes occurred, suggesting an anti-inflammatory mechanism. The drug was well tolerated and showed sustained effects [165].

The oral NLRP3 inhibitor dapansutrile (OLT1177) is being evaluated in a phase 2 trial in patients with mild inflammatory T2DM (DAPAN-DIA, NCT06047262), with endpoints including glycemic control, microvascular complications, and metabolic parameters [166]. Prior studies reported good tolerability, with high doses (1,000 mg/day) causing mild to moderate gastrointestinal discomfort and metabolic abnormalities [167,168]. No renal-specific clinical data are available yet. Another NLRP3 inhibitor, MCC950, showed renoprotective effects in animal models but has not progressed to clinical trials due to hepatotoxicity concerns [169]. In addition, there are numerous other emerging or potential therapies for tubulointerstitial inflammation, which are described in Table 3. Inhibitors targeting the NLRP3 inflammasome (e.g., CY-09 and CAY10603) and Toll-like receptor signaling (e.g., CRX-526) have been demonstrated to attenuate tubulointerstitial inflammation in preclinical models, supporting their relevance as upstream modulators of inflammatory activation [94,170,171].

**Table 3.** Emerging or potential therapies targeting tubulointerstitial inflammation.

Intervention	Categories	Mechanism of action	Drug	Outcome	Main side effects	phase
Targeted Therapy for Classic Inflammatory Signals	NLRP3 inhibitor	NLRP3	MMC950	Alleviated renal dysfunction and tubulointerstitial injury	Hepatotoxicity	Preclinical study
			CY-09	Alleviated tubulointerstitial injury	NR	Preclinical study
			CAY10603	Alleviated renal dysfunction and reduced macrophage infiltration, tubular injury and tubulointerstitial fibrosis	NR	Preclinical study
	TLR4 inhibitor	TLR4	CRX-526	Albuminuria↓ macrophage infiltration↓	NR	Preclinical study
Senotherapeutics	Senolytics	Eliminating senescent cells that resist apoptosis	Dasatinib and Quercetin	Circulating SASP Factor↓ Organize CD68⁺ macrophages↓	Potential nephrotoxicity	Phase 1 pilot study (NCT02848131)
			fisetin	Alleviated tubulointerstitial injury	NR	Preclinical study
	Senomorphics	Regulating SASP	Rapamycin	Alleviated tubulointerstitial injury	NR	Preclinical study
Targeted Therapy for microRNA	MiRNA	Silence MiR-21	Antagomir-21	Improved Renal function and ACR	NR	Preclinical study
		Silence MiR-20a	Target-based (no approved drug)	Alleviated tubulointerstitial injury	NR	Preclinical study

*Abbreviation:* NR, not reported.

Although early clinical trials targeting individual inflammatory pathways have provided important proof-of-concept evidence, their overall efficacy remains limited by pathway redundancy, disease heterogeneity, and the lack of tools capable of capturing the dynamic and spatial features of tubulointerstitial inflammation (TII). These constraints have stimulated increasing interest in next-generation translational strategies that extend beyond single-pathway inhibition.

Multi-target nanotherapeutic approaches represent a promising strategy for simultaneously modulating inflammatory and pro-fibrotic programs while enhancing renal regional specificity. Recent studies demonstrate that biomimetic lipid nanoparticles can preferentially target injured tubular epithelial cells, for example through kidney injury molecule-1 (KIM-1)–mediated uptake, enabling the co-delivery of anti-inflammatory and anti-fibrotic small molecules. This strategy reduces inflammatory cytokine expression, limits immune cell infiltration, and remodels the fibrotic microenvironment in chronic kidney disease (CKD) models [172]. Other kidney-targeted platforms have achieved combined delivery of small molecules and siRNA to tubular epithelial cells, highlighting the practical feasibility of multi-payload therapeutic designs in renal disease [173]. Although direct evidence for dual siRNA delivery targeting multiple inflammatory pathways (e.g., NLRP3 and CCL2) in nephropathy remains limited, *in vivo* studies confirm selective uptake of siRNA nanoparticles by proximal tubules *via* megalin-mediated endocytosis, supporting the methodological feasibility of multi-target RNA-based therapeutics for future TII-focused interventions [174].

Therapies targeting senescent cells, including senolytic agents and strategies that modulate the senescence-associated secretory phenotype (SASP), have emerged as a novel avenue for treating chronic tubulointerstitial inflammation. Increasing evidence indicates that senescent tubular epithelial cells and their SASP sustain persistent immune activation and promote a pro-fibrotic microenvironment. A combination of dasatinib, a second-generation tyrosine kinase inhibitor, and quercetin, a plant-derived flavonoid with senolytic activity, has demonstrated renoprotective effects in multiple experimental models [175]. In diabetic nephropathy, this combination improves renal function and attenuates histological injury, including reductions in interstitial fibrosis and glomerular basement membrane thickening. These effects are associated with enhanced autophagy and suppression of tubular senescence, as reflected by decreased expression of p16, p53, and fibronectin [175]. The translational potential of this approach is currently under investigation in an open-label phase I pilot trial (NCT02848131) enrolling elderly patients with type 2 diabetes and CKD (eGFR 15–45 mL/min/1.73 m^2^) [176]. Preliminary findings indicate reduced expression of senescence markers (p21 and p16), decreased senescent cell burden, lower circulating SASP factors, and diminished renal CD68^+^ macrophage accumulation [176], supporting the concept that clearance of senescent cells may alleviate chronic inflammatory signaling. Nevertheless, clinical translation warrants caution. Although early studies have not revealed overt nephrotoxicity, dasatinib has been associated with rare renal adverse events, including proteinuria and podocyte injury, in real-world settings, underscoring the need for systematic evaluation of safety, off-target effects, and optimal dosing in CKD populations [175].

Similarly, fisetin, another flavonoid compound, has been shown to reduce senescent tubular epithelial cell burden, suppress SASP expression, and ameliorate tubular injury in experimental models of diabetic nephropathy and lupus nephritis. Chronic kidney disease models further demonstrate its capacity to attenuate inflammation and interstitial fibrosis associated with tubular damage [175,177,178]. In parallel, mechanistic target of rapamycin (mTOR) inhibitors, such as rapamycin and sirolimus, have long been recognized for their protective effects against tubulointerstitial injury in obstructive nephropathy, with reductions in inflammatory cell infiltration and extracellular matrix deposition linked, at least in part, to SASP modulation [179,180]. In addition, nicotinamide and other NAD^+^-enhancing strategies exhibit anti-inflammatory and anti-fibrotic effects across multiple models of renal interstitial fibrosis, providing a complementary approach to modulating chronic inflammation through the metabolic–aging axis, although evidence remains largely preclinical [181].

Cell-based therapies and extracellular vesicle (EV)/exosome-based approaches are also undergoing rapid evolution, shifting away from unselective mesenchymal stem cell infusions toward more controlled and engineered payload delivery systems. In unilateral ureteral obstruction models, engineered mesenchymal stem cells overexpressing specific microRNAs, such as let-7c, transfer these miRNAs to injured tubular epithelial cells *via* exosomes, thereby attenuating renal injury and fibrosis [182]. Similarly, EVs carrying anti-inflammatory miRNAs, including miR-181d, suppress pro-inflammatory signaling pathways such as NF-κB and protect against renal fibrosis [183]. Additional studies demonstrate that miR-21 and miR-20a reduce tubulointerstitial inflammation in diabetic nephropathy models [184,185]. Collectively, these findings support the feasibility of engineering mesenchymal stem cell–derived exosomes to deliver anti-inflammatory miRNAs that target immune–tubular epithelial crosstalk in TII.

Finally, artificial intelligence–guided drug repurposing based on transcriptomic reversal algorithms provides an efficient complement to conventional target-based drug discovery. Connectivity mapping approaches, such as CMap and LINCS, leverage large-scale drug-induced gene expression atlases to identify compounds predicted to reverse disease-associated transcriptional signatures. In CKD, connectivity mapping of a progression-associated gene signature identified vorinostat as a candidate therapeutic agent, with subsequent *in vivo* validation demonstrating attenuation of tubulointerstitial fibrosis and improved survival in experimental models [186]. Similar LINCS-based strategies in diabetic kidney disease have identified and experimentally validated additional candidates, including BI-2536 [187], illustrating a scalable translational pipeline from omics-based disease signatures to in silico drug prioritization and preclinical validation.

## 7. Future prospects

The progression of glomerular diseases involves not only intrinsic glomerular injury but also secondary Interstitial inflammation, which significantly contributes to chronic kidney damage. Evidence indicates that interstitial inflammation amplifies glomerular injury signals and promotes irreversible parenchymal remodeling through immune cell infiltration, inflammatory cascades, and pro-fibrotic signaling. Clinicopathological studies confirm that the severity of interstitial inflammation predicts the risk of end-stage renal disease (ESRD). Although its prognostic value is increasingly recognized, only IgA nephropathy, Lupus nephritis, and BAFF scoring currently incorporate interstitial inflammation in evaluation systems – other glomerular diseases have yet to follow. Further research is needed to validate the prognostic impact of interstitial inflammation across different nephropathies.

First, although substantial evidence supports the prognostic relevance of tubulointerstitial inflammation (TII), its clinical interpretation remains heterogeneous. Variability in scoring systems, sampling regions, and covariate adjustment strategies has resulted in inconsistent estimates of its independent predictive value across different disease entities. Future studies should therefore prioritize refinement of how interstitial inflammation is quantified and interpreted, moving beyond categorical or semi-quantitative descriptors toward continuous and reproducible metrics that more accurately capture inflammatory burden, spatial heterogeneity, and its interaction with fibrotic remodeling.

Second, while targeted anti-inflammatory strategies – including inhibition of the JAK/STAT, CCL2/CCR2, and NLRP3 pathways – have demonstrated promise in early-phase studies, robust clinical validation remains limited. Most trials rely on proteinuria reduction or histological endpoints, which may incompletely reflect changes in interstitial immune activity. Larger, adequately powered studies with longer follow-up and improved patient stratification are required to determine whether modulation of interstitial inflammation confers sustained renal protection. In parallel, the development of therapeutic approaches targeting chronic inflammatory persistence, immune–stromal crosstalk, and inflammation–fibrosis coupling remains a critical unmet need.

A major barrier to progress in this field is the lack of sensitive and specific noninvasive tools for monitoring TII dynamics. The inability to repeatedly assess interstitial immune activity *in vivo* limits patient selection, treatment response evaluation, and early identification of irreversible injury. Accordingly, future efforts should focus on the development and validation of noninvasive biomarkers derived from urine and blood, advanced imaging modalities, and integrated biomarker panels that reflect interstitial immune activation rather than glomerular injury alone.

Beyond individual biomarkers, a more comprehensive characterization of the interstitial immune microenvironment is required. Recent advances in spatial transcriptomics, single-cell profiling, and AI-augmented digital pathology now enable high-resolution dissection of immune–tubular–stromal interactions. An important next step will be the integration of these data into predictive frameworks capable of modeling disease trajectories, particularly the transition from potentially reversible inflammation to established fibrosis. In this context, AI-driven systems approaches – such as digital twins or agent-based models incorporating spatial immune architecture, cytokine signaling, and fibrosis kinetics – may help identify critical thresholds beyond which inflammatory processes become self-sustaining and fibrogenesis irreversible.

Building on these methodological advances, a future-oriented objective is the development of an integrated precision framework for TII assessment. One potential approach is the establishment of a Renal Inflammation Precision Index (RIPI), integrating (i) AI-quantified digital histopathology capturing spatial patterns of inflammation, (ii) noninvasive biomarker panels reflecting immune activation and tubular injury, and (iii) molecular pathway activation scores derived from multi-omics analyses. Such an index could provide a standardized tool for patient stratification, enrichment of clinical trial cohorts, and longitudinal monitoring of therapeutic responses in interstitial inflammation–targeted interventions.

Finally, the implementation of data-intensive, AI-enabled precision strategies raises important ethical, regulatory, and governance challenges. Multi-center data sharing is essential for robust model development and validation but must be balanced against patient privacy and data security considerations, potentially through federated learning or privacy-preserving analytic frameworks. In parallel, regulatory pathways for digital biomarkers and AI-derived endpoints in inflammatory kidney disease remain insufficiently defined. Early engagement with regulatory agencies will be necessary to establish standards for analytical validity, clinical relevance, and reproducibility.

## Data Availability

Data sharing is not applicable to this article as no data were created or analyzed in this study.
